# Parasitic Effects on Electrical Bioimpedance Systems: Critical Review

**DOI:** 10.3390/s22228705

**Published:** 2022-11-11

**Authors:** David William Cordeiro Marcôndes, Aleksander Sade Paterno, Pedro Bertemes-Filho

**Affiliations:** Center for Science and Technology, Department of Electrical Engineering, Santa Catarina State University, Joinville 89219710, Brazil

**Keywords:** electrical bioimpedance, error compensations, parasitic capacitances, critical review

## Abstract

Parasitic capacitance represents the main error source in measurement systems based on electrical impedance spectroscopy. The capacitive nature of electrodes’ impedance in tetrapolar configuration can give origin to phase errors when electrodes are coupled to parasitic capacitances. Nevertheless, reactive charges in tissue excitation systems are susceptible to instability. Based on such a scenario, mitigating capacitive effects associated with the electrode is a requirement in order to reduce errors in the measurement system. A literature review about the main compensation techniques for parasitic capacitance was carried out. The selected studies were categorized into three groups: (i) compensation in electronic instrumentation; (ii) compensation in measurement processing, and (iii) compensation by negative impedance converters. The three analyzed methods emerged as effective against fixed capacitance. No method seemed capable of mitigating the effects of electrodes’ capacitance, that changes in the frequency spectrum. The analysis has revealed the need for a method to compensate varying capacitances, since electrodes’ impedance is unknown.

## 1. Introduction

The COVID-19 outbreak accounted for thousands of deaths between 2019 and 2020. Despite its low mortality rate, its high transmission rate and associated breathing complications have led to the crowding of health systems worldwide, and illuminated the logistical difficulties in the acquisition and use of medical equipment. Many mechanical ventilators and devices were built to follow-up the clinical frame of infected patients’ respiratory tracts. Given such a high medical demand during the pandemic, countries around the world [1,2,3,4] have turned their private manufacturers and industrial facilities into assembly lines for such class of equipment. Accordingly, fast, low-cost and robust medical resources became essential to save lives throughout epidemic breakouts. Lung activity monitoring through the electrical impedance tomography method (EIT) is well-known in the literature [5,6,7]. This method is viewed as a feasible alternative to X-ray imaging due to its low cost, robustness and easy implementation [8,9]. Modern CT scanners can track a patient’s real-time respiratory evolution and frequency [10,11], which presents a challenging task to accomplish with an X-ray machine. Electrodes are fixed on the patient’s thorax in EIT systems, where these electrodes are then used to transfer an electrical current. Subsequently, the resulting power around all electrodes is measured. Therefore, it is regarded as a non-invasive diagnostic tool that does not expose monitored patients to either ionizing radiation or harmful substances, despite their physiological conditions [12]. EIT technologies are based on impedance distribution in biological tissues that, in turn, show anisotropic behavior towards different frequencies, given their specific dispersion under a certain electromagnetic field [13]. Tissues are excited through electrodes fixed on their surface. The choice for the type and number of electrodes is crucial for both excitation and measuring responses to the electromagnetic field [14].

With respect to bioimpedance measurement in tissue, it is possible to employ two, three or four electrodes in the excitation and measurement procedure. The four-electrode arrangement is more often used in bioimpedance spectroscopy [13]. This configuration is adopted in an attempt to rule out the effects of both polarization and electrode/tissue interface impedance, which varies depending on the electrode configuration [14]. Therefore, the electrodes’ effects must be mitigated as much as possible in measurements that bear real impedance value in the deepest tissues of patients [15]. The system known as electrical impedance tomography (EIT) enables diagnosis in several medical applications based on the image of the aforementioned distribution, since the identification of certain pathology or medical conditions would be effective in this case [16].

There are two possible configurations for the four-electrode/wire measurement system, also known as tetrapolar measurement [17,18,19], namely: (i) voltage-controlled current source (VCCS) to excite tissues through a pair of electrodes, while an instrumentation amplifier measures voltage drop, which leads to another different pair of electrodes; (ii) voltage-controlled voltage source (VCVS) to excite tissues through a pair of electrodes, while the other pair of electrodes are used to measure the current through a current/voltage converter. The pair of electrodes in the second configuration must share a common reference, because predicting current distribution in the tissue [20] is a task of higher complexity. However, the first configuration has the advantage of decoupling the electrode pairs and enabling the use of multiplexers. Thus, the VCCS method is more often applied to electrical impedance tomography systems [21].

However, parasitic capacitances in real tetrapolar systems used in EIT compromise the measurements [16,22]. Given the environment, cables and connections, these capacitances form a pathway [23], so that the leakage current overestimates tissue impedance as the frequency increases [24]. Measurement equipment capacitances are coupled to other capacitances that are generated by the electrolyte’s ionic distribution at the electrode/tissue interface. Such a coupling allows leakage currents to the ground, thus compromising the impedance spectrum [25]. It is not always possible to calculate the value of this capacitance, even if one uses the approximations in previously determined frequencies, given the electrode capacitance’s dependence on potential and excitation frequency [26,27]. Furthermore, the current source impedance is sensitive to leakage capacitance at high frequencies [28]. Therefore, techniques used to reduce electrode capacitance can help tissue decoupling in studies on often-fixed measurement systems, and systematic errors may be avoided by using electrode positioning protocols [29].

The literature presents several instrumentation systems based on capacitance compensation techniques for bioimpedance. These can be divided into three large groups, according to the adopted configuration’s operation [21,22,30,31,32,33]: (A) compensation in instrumentation; (B) compensation in measurement processing; (C) compensation based on negative impedance converters. All herein analyzed and catalogued articles have proposed compensation techniques or strategies to mitigate the effects of fixed and previously known parasitic capacitance, i.e., capacitance value is assumed constant during measurement experiments.

## 2. Electrode Capacitance Effects

**Figure 1:**
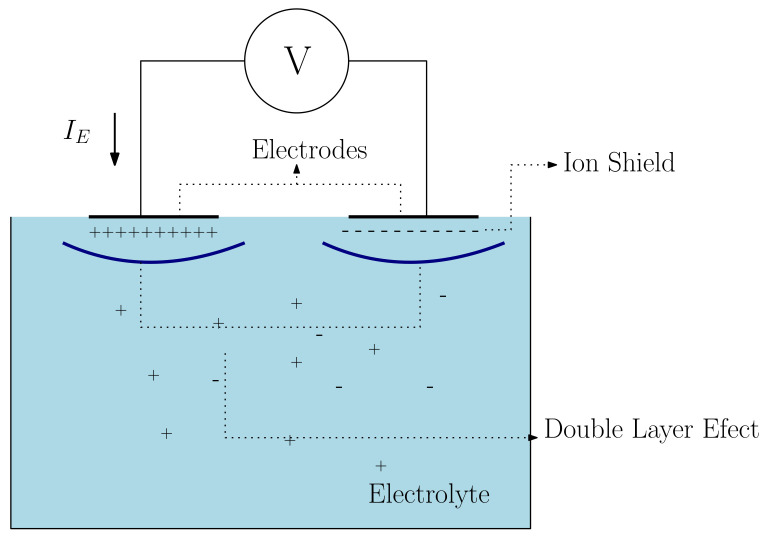
Simplified impedance measurement system of a homogeneous electrolyte.

The model in Figure 1 simplistically and ideally shows the physical processes involved at the electrode/tissue interface. The current $I_{E}$ in the electrodes represents the transport of charge and ions within the electrolyte (tissue). Although certain body tissues and fluids, such as blood, can be approximated by the electrolytic cell model with reasonable accuracy, experimental results cannot be explained by this model at high frequencies [13]. Based on this model, a homogeneous and isotropic distribution of ions and charges in the electrolyte can be assumed without the presence of electrodes. However, the redistribution of charges and ions occurs in the presence of electrodes, even if they are chemically inert and subject to a null power (short circuit between electrodes). An ionic layer forms over the electrode’s surface due to differences in electronegativity between the electrolyte and the electrode [13].

With respect to the low-intensity power, the electrolytic model can be approximated by a parallel system formed by a resistor and a capacitor [34]. Resistance arises from the ionic displacement of charges at the electrode/electrolyte interface. Ions are attracted from the electrolyte and deposited on the electrode’s surface—this phenomenon is known as Helmholtz’ double layer effect [35,36,37]. This ionic layer forms a shield against the flow of new charges that can be displaced due to microscopic irregularities on the electrode’s surface. Therefore, the shield forms a dielectric that is subjected to the power generated by ionic redistribution (polarization potential), with the externally applied one (V). The van der Waals intermolecular force accounts for the attraction of nearer molecules arising from the polar interactions of the induced dipole type [38]. The voltage on the electrode’s surface decreases as *V* increases. The van der Waals interaction mitigates the strength of the intermolecular attraction as the electrostatic repulsion between ions increases due to the increased concentration of ions bearing the same polarity on the electrodes’ interface [34].

The van der Waals interaction accounts for the work necessary to separate two neighboring molecules, a fact that results in superficial mechanical voltage. The *V* potential displaces charges presenting the same signal towards the electrode, and it causes the Coulombic repulsion to reduce the work necessary to separate the molecules, a fact that reduces the superficial voltage [34]. Accordingly, $V_{ZTS}$ is defined as the electrodes’ potential when superficial voltage is null.

More ions migrate from the electrolyte to the electrode in order to achieve small increase in *V*, in $V_{ZTS}$’ surroundings, and proportionately increases the ionic shield layer thickness. The capacitance seen through the electrodes decreases when there is no ionic absorption or desorption reactions between ions, i.e., if there is no variation in the chemical potential [34]. This effect is described by Equation (1); wherein the associated capacitor is known as Helmholtz’s capacitance ($C_{H}$) [13] and $q_{m}$ represents the mean value of the charge inside the *V* interval, which is marginally higher than that of $V_{ZTS}$. $C_{H}$ can reach hundreds of nF in typical configurations [13]. It is worth noting that even with fluctuations in the chemical potential and in $V\gg V_{ZTS}$, capacitance caused by the accumulation of ions on the electrode’s surface still influences the measurement system [34]. Limiting the size of the electrode’s cross-sectional area is an alternative to reduce the aforementioned effects [14]. However, this process can increase impedance at the electrode/electrolyte interface, which, in turn, poses project limitations to electronic instrumentation [34].

(1) $$C_{H}=\frac{q_{m}}{V-V_{ZTS}}$$

**Figure 2:**
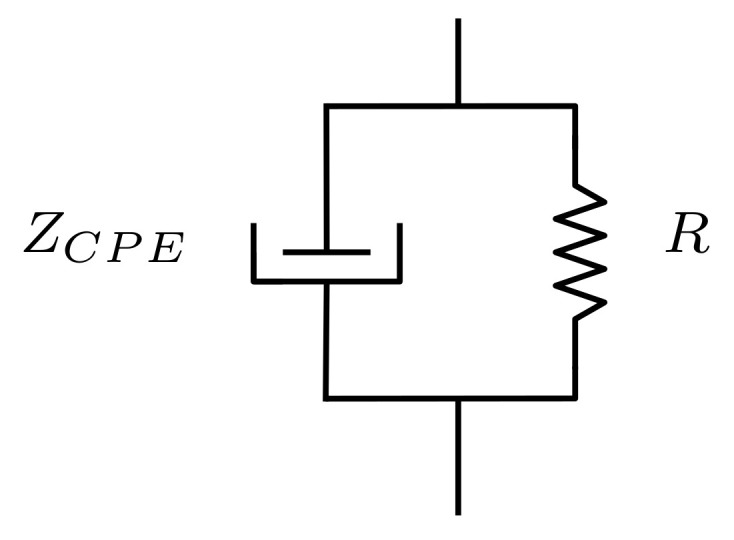
Electric equivalent of electrode impedance based on the element of the constant phase $Z_{CPE}$.

Chemical reactions significantly change the simple electrolyte model in biological tissue [34]. An increase in the electrode potential under an excitation frequency is followed by phenomena such as dielectric constant dispersion, ionic disruption and variation in charge mobility [34]. Accordingly, in order to better represent experimental data, the electrode/tissue interface is better described by the model shown in Figure 2. Ionic current transport in this model is represented by the *R* resistor, whereas electrolytic effects are represented by the element of the $Z_{CPE}$ constant phase element. Experiments have highlighted that the impedance’s real and imaginary components have constant phase in specific regions of the spectrum, which are known as dispersion zones—whenever there is no ionic current present (R→∞) [39]. Therefore, the phase is described by element $Z_{CPE}$, so that:

(2) $$Z_{CPE}=\frac{K}{{\left(j\omega \right)}^{\beta }}$$

In Equation (3), K,ω,β,j are the stationary impedance, angular frequency, dispersion factor and imaginary operator ($j=\sqrt{-1}$), respectively.

The constant *K* of biological tissues depends on the electrodes’ voltage [34]. From a capacitive reactance viewpoint, $Z_{CPE}$ can be understood as a capacitor, whose value is the function of the electrode’s power and of excitation frequency, so that:

(3) $$C_{CPE}=\frac{1}{K}{\left(j\omega \right)}^{\beta -1}$$

In fact, if one assumes perfectly polarizing electrodes (R→∞), the C capacitance, seen through the electrodes, depends significantly on the superficial voltage at the electrode/tissue interface [34], so that:

(4) $$C=-\frac{\partial^{2}\zeta }{\partial V^{2}}$$

Wherein ζ is the superficial voltage.

Equation (4) relates capacitance to mechanical elements in a geometric manner based on the electrodes’ special arrangement [40]. This means that in the case of a regime where *C* variation is approximated as proportional to ζ, the electrode’s capacitance can face sinusoidal variations in relation to *V*, because the solution of the differential Equation (4) bears a sinusoidal shape. Therefore, electrode capacitance demonstrates non-linear behavior as a function of the voltage *V*. Although the reduction in the contact field with the tissue also reduces *C*’s magnitude, its variation during the spectral measurement process remains the same. Not knowing capacitance at every instant leads to difficulty in compensating the leakage current in electrodes [28]. When *C* couples to parasitic capacitances between the tissue/electrode interface and the ground, a leakage current introduces errors in the measurement system that seem to result from the assessed tissue’s impedance. Authors in [41] reported this problem when they found an impedance discrepancy in the same experiment, although exposed in different environments.

## 3. Parasitic Capacitance in Measurement Systems

EIT measurement systems are susceptible to influence from several external sources. The current source used to excite tissues in tetrapolar systems is sensitive to the source’s output capacitance in relation to the ground. If the measurement equipment is properly grounded, then the assessed biological sample environment shares the same power (ideally), which is the measurement system’s reference [13]. When there is an imbalance in ground power between the measurement system and the assessed sample (for example, a patient), and lack of Ohmic connection, then subsequent power fluctuations will generate a leakage current through parasitic capacitance occurring between the grounding system and the electrode in contact with the patient. Nevertheless, a group of finite conductors *j* subject to a potential $V_{j}$ (in relation to the finite one) are mutually coupled in the same absolute vacuum— independent from the ground—so that:

(5) $$Q_{i}=C_{i}V_{i}+\sum_{i\neq j}C_{i,j}V_{i,j}$$

wherein $Q_{i}$ is the charge in conductor *i*, $V_{i,j}$ is the variation in the voltage *i* in relation to conductor *j* and $C_{i,j}$ is the so-called mutual-capacitance of conductor *i* [42].

Therefore, conductors in close proximity to electrical wires and cables, even in other equipment, are susceptible to the parasitic capacitance that couples them. The electrostatic shield does not reduce the coupling effects shown in Equation (5), because the dynamic variation of potential $V_{i,j}$ also accounts for coupling between conductors. Therefore, it is possible to limit fast transitions in conductor potential to mitigate the $C_{i,i}$ value by decreasing the excitation frequency. Imposing $V_{i,j}=0$ for a pair of any conductors also decreases coupling. If the internal shield loop shares the same power as the core conductor’s signal in a three-way coaxial cable, for example, the mutual capacitance between the conductor’s signal and the external loop is null. However, parasitic capacitance between the patient and the grounding is connected in series with the electrodes’ capacitance at the tip of the coaxial cable [28]. Owing to the varying nature of the electrodes’ capacitance, the total capacitance seen through the ground source can only be understood as constant within both a low frequency approximation and small signals. In this case, by changing the electrodes’ position on the patient’s body, for example, the output parasitic capacitance can be altered [28].

Capacitors coupled to the exit of a current source limit the operation frequency. The rate of change in current *I*, which is applied to a fixed-value capacitor, is proportional to the second time derivative of voltage *V*, over the capacitor. The $d^{2}V/dt^{2}$ is proportional to frequency in Laplace’s domain, whereas dI/dt is proportional to the square of the frequency. As a consequence, the source exit must be capable of carrying the voltage even faster than variations in the current, in order to follow-up the current variation over the capacitive charge. Charges stored in chip capacitors oppose current variation [43]. However, Howland current sources [44] are built with operational amplifiers (OpAmp) whose maximal output voltage variation is limited by OpAmp’s slew rate (SR) [43]. The higher the OpAmp gain–bandwidth product, the higher its SR, and the better the performance of the current source with capacitive charges [45].

Capacitive charges can affect the stability of the current source. The capacitive charge is coupled to its output impedance in a typical Howland current source; this output, in turn, adds a pole to the source transference function. If the gain and stability margin do not embody the additional phase delay due to the additional pole, i.e., if the location of this pole is not known given the unawareness about the capacitance, the current source can thus oscillate or saturate its output. A dominant pole must be added to the system to ensure that phase displacement does not exceed 180 degrees before a drop in the closed-loop unitary gain of the source, in order to solve this issue. However, the pole’s position equally reduces the current source’s operation band [46]. Finally, the parasitic capacitances on the source output have to be mitigated in order to improve the performance of the current source. The literature provides more robust current source configurations concerning reactive charges, such as the case of the modified and mirrored Howland current sources [47].

## 4. Parasitic Capacitance Reduction Techniques

Most studies on parasitic capacitance reduction techniques are found in scientific journals and in the proceedings of events in the biomedical engineering field. The employed methods were grouped into three categories based on a systematic search for articles published in the literature in recent decades; moreover, the advantages and disadvantages regarding their implementation are also discussed.

### 4.1. Compensating the Voltage Measured in the Charge

Impedance of the electrodes used to measure the resulting power is also affected by the parasitic impedance in a tetrapolar system excited by a current source. Instrumentation with high-input impedance is necessary to reduce the contact-resistance effects of electrodes and/or connections. *R* represents the flow of charges and ions that effectively circulate in tissues’ deepest layers (Figure 2) [34]. This impedance originates from charges crossing the ionic shield formed by Helmholtz’s effects. The electrode/tissue contact field favors the ionic migration of the shield layer. The associated transport of charges is related to the reduction of the effective *R* value in water medium with higher concentration of free ions. In experimental terms, dry electrodes’ impedance can be up to three times higher in magnitude than that recorded for wet electrodes, which may reach dozens of MΩ [48].

**Figure 3:**
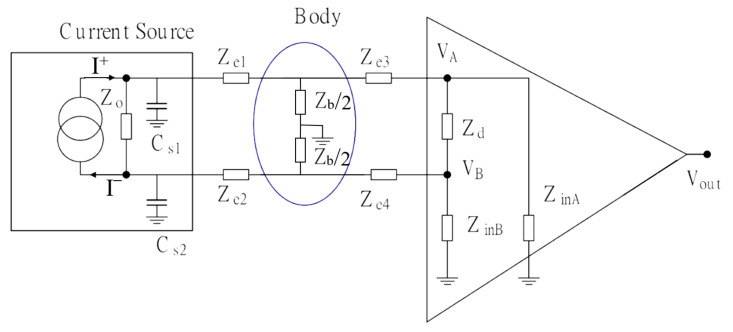
Parasitic impedance of the excitation and measurement system, where $V_{out}=G$. $\left(V_{A}-V_{B}\right)$ and *G* is the voltage gain of the instrumentation amplifier.

The input impedance of the electronic instrumentation linked to the reading electrode forms an electrical voltage divider along with a signal in the assessed sample. Consequently, there is a reduction in intensity of the signal to be measured. Figure 3 shows the tetrapolar measurement system coupled to electronics’ non-idealities. The output impedances $Z_{o}$ of the current source *I* and both differential input $Z_{d}$ and common inputs ($Z_{inA}$ and $Z_{inB}$) of the instrumentation amplifier (IA) mitigate the signal, mainly at high frequencies. Similarly, the high-output impedance ($Z_{o}$) of the current source is also susceptible to errors in the presence of parasitic impedances ($C_{s1}$ e $C_{s2}$). If one takes into account the circuit in Figure 3, the voltage at terminal *A* of the instrumentation amplifier is influenced by impedances and parasitic capacitances of the set of circuits, so that:

(6) $$V_{A}=\frac{\left(Z_{b}^{{}^{\prime }}\right)(Z_{e3}+Z_{input}\left|\right|Z_{d}^{{}^{\prime }})}{Z_{b}^{{}^{\prime }}+Z_{e3}+Z_{input}\left|\right|Z_{d}^{{}^{\prime }}}\cdot \frac{Z_{o}^{{}^{\prime }}\left|\right|X_{Cs1}I^{+}}{Z_{e1}+(Z_{o}^{{}^{\prime }}\left|\right|X_{Cs1})}$$

where $Z_{d}^{{}^{\prime }}=Z_{d}/2$, $Z_{b}^{{}^{\prime }}=Z_{b}/2$, $Z_{o}^{{}^{\prime }}=Z_{o}/2$ and $Z_{inA}=Z_{inB}=Z_{input}$.

By assuming a balanced current source (a balanced current source assumes that the amplitude of the source side $I^{+}$ is equal to that at the sink side $I^{-}$), an instrumentation amplifier with balanced inputs $Z_{input}>>Z_{e}$ ($Z_{e}$ = $Z_{e1}$ = $Z_{e3}$) and $Z_{o}>>Z_{e}$, then the voltage in the *A* terminal of the amplifier is given by:

(7) $$V_{A}=\frac{Z_{b}\cdot I^{+}}{\left(2+Z_{b}/Z_{input}\right)\cdot \left(1+SC_{s1}\right)}$$

The input impedance $Z_{input}$ is provided by the amplifier’s manufacturer with regard to a given resistance $R_{input}$ that is in parallel to a capacitance $C_{input}$. In this case, if one takes into consideration that $R_{input}>>Z_{b}$, Equation (7) can be rewritten as in Equation (8):

(8) $$V_{A}=\frac{Z_{b}\cdot I^{+}}{\left(2+SC_{input}\cdot Z_{b}\right)\cdot \left(1+SC_{s1}\right)}$$

One can observe that parasitic capacitance of both the instrumentation amplifier and the current source generates poles that mitigate output voltage $V_{out}$ of the measurement system, mainly at high frequencies, leading to the generation of undesirable variations.

**Figure 4:**
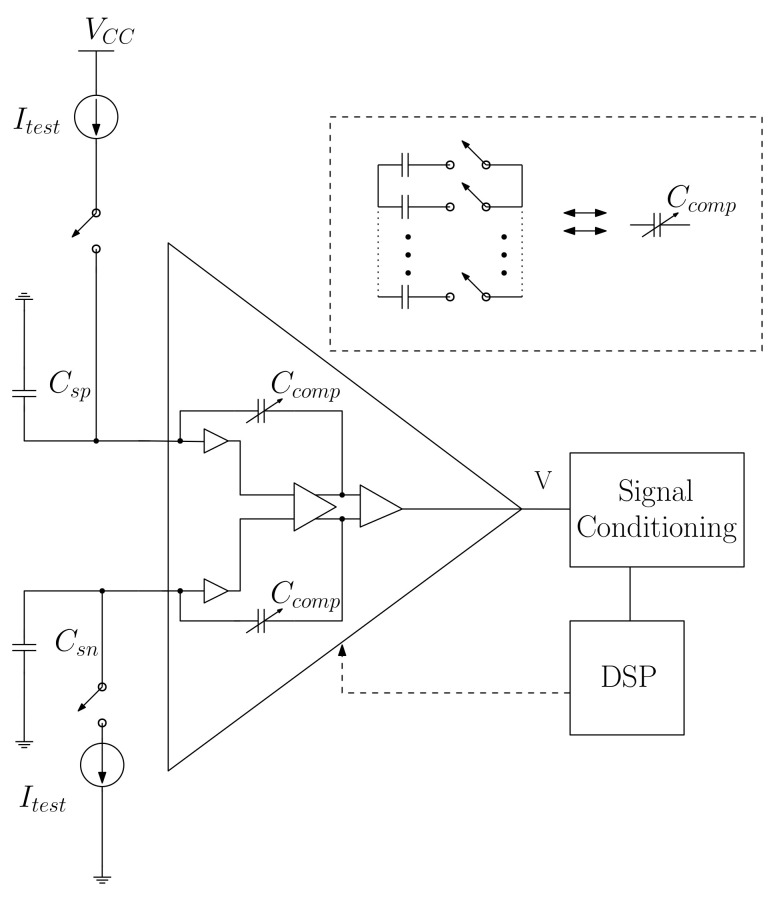
Internal compensation to the instrumentation amplifier. $C_{sp+},C_{sp-}$ are the parasitic capacitances observed in the instrumentation amplifier’s input nodes. $I_{test}$ is the current source used to calibrate the compensation system, $C_{comp}$.

However, if the leakage current value at the terminals ($V_{A}$ and $V_{B}$) of the instrumentation amplifier is known, then, the variation in the instrumentation amplifier can be compensated. This compensation technique is adopted by the authors in [49,50]. Author [49], in particular, describes compensation by using an internal node of the instrumentation amplifier, whose input has parasitic capacitances $C_{sp}$ and $C_{sn}$, as shown in Figure 4.

The idea proposed by [49] introduces an adjustable capacitor $C_{comp}$ in the internal nodes of the instrumentation amplifier. Appropriate internal nodes are selected to insert the compensation loop by analyzing the small signals model of the instrumentation amplifier.

Capacitor $C_{comp}$ is introduced in the node to form a feedback loop that generates a negative value capacitor by Miller approximation in the inputs [43]. A whole set of analog switches allows for the varying of $C_{comp}$ in $2^{8}$ discrete capacitance intervals. Current sources $I_{test}$ are connected to inputs during the calibration stage and to a digital controller that adjusts $C_{comp}$ to the maximum dV/dt. This method enables increasing impedance in the system; thus, it compensates for the leakage current through input parasitic capacitance. $C_{comp}$ introduction is known as negative capacitance compensation in positive and negative inputs that, in turn, present a linear approximation at low frequencies.

Among its advantages, one can note:

- Using the installed instrumentation’s electronics by discarding the use of new operational amplifiers in the measurement system and by reducing the area and the production costs of the integrated circuit;
- Compensating for the system’s low influence (<2%) in the amplifier’s Power Source Rejection Ratio (PSRR), Common Mode Rejection Ratio (CMRR) and Total Harmonic Distortion (THD). In addition, open loop gain and frequency band remain unchanged;
- Increasing the input impedance by two decades in the kHz range [49], considering that it can reach 100 GΩ at low frequencies;
- Reducing power consumption of the amplifier.

On the other hand, disadvantages of this compensation type are:

- This compensation model disregards the intrinsic capacitances of transistors. These capacitances reduce the transconductance characteristics of the circuit at relatively high frequencies (10 kHz);
- Temperature drift affects the open-loop gain of the OpAmp due to internal changes in the transistors’ transconductance. Furthermore, MOS transistors’ transconductances are a function of the drain current [43], which subsequently influences the open-loop gain of the OpAmp. These changes affect the linearity of the compensation capacitance $C_{comp}$, which impedes full compensation for a wide range of parasitic capacitances;
- Influence from the output impedance of $I_{test}$ sources. The impedance of sources during the calibration stage can introduce over/under compensation for parasitic capacitances given that the input impedance magnitude is within the *G*Ω range.

### 4.2. Excitation Current Compensation

EIT analyses are carried out through tissue conduction models, such as the Cole–Cole model [13]. However, parasitic capacitances introduce errors to experimentally measured variables. Systematic errors caused by leakage currents can be compensated throughout the signal’s analog processing stage if the measurement system is properly calibrated and known [41,51]. In addition, one would also observe in some situations at high frequencies, in a range reaching the usable limit of 1 MHz in EIT systems, certain effects that could be interpreted as parasitic effects of an interplay of inductive and capacitive nature [52].

One must take into consideration the measurement system shown in Figure 3 in order to understand the compensation mechanisms. If one assumes a high-output impedance current source, high-input impedance instrumentation amplifier and $Z_{e1}$ = $Z_{e2}$ = $Z_{e3}$ = $Z_{e4}$ = 0, the impedance measured at a certain location of the body is given by:

(9) $$Z_{meas}=\frac{V_{b}\left(1+SC_{s}\right)}{I}$$

where $I=I^{+}=-I^{-}$, which, in this case, refers to a balanced current source, i.e., the current of source $I^{+}$ has magnitude equal to that of the current of drain $I^{-}$.

The current diverted from the analyzed body tissue increases with frequency. By assuming a parasitic capacitance $C_{s}$ of 10 pF, one can calculate the measured impedance in the order of 0.2 ppm, at a frequency of 10 MHz, which is higher than the true value. As the parasitic capacitance increases, so does the impedance calculation error, even at lower frequencies. Therefore, the leakage current has the effect of overestimating the true $Z_{b}$ value at high frequencies. The $C_{input}$ capacitances of the instrumentation amplifier are not taken into consideration in this analysis.

The reactive nature of the $I_{stray}$ parasitic current leads to errors in the module and impedance phase $Z_{b}$ [41]. However, if the value associated with the parasitic impedance $C_{s}$ is known, then, the $I_{stray}$ contribution value in $Z_{b}$ can be analytically removed. This method consists of multiplying the $Z_{meas}$ impedance by a phase factor:

(10) $$e^{-j\omega TD(\omega )}$$

where TD(ω) is the phase delay term triggered by $C_{s}$. By applying the natural logarithm in the phase factor (10) and by equating it to the product with the phase factor in Equation (9), $Z_{b}$ can be determined by:

(11) $$\begin{array}{ccc} Z_{b} & =Z_{meas}e^{-j\omega TD(\omega )} & \\ TD(\omega ) & =\frac{ln(1-j\omega Z_{meas}C_{s})}{j\omega } \end{array}$$

Thus, this method effectively allows for the removal of the $I_{stray}$ contribution as long as the parasitic capacitance value is accurately known. It is worth noting that the measurement system can become susceptible to errors when one estimates the $Z_{meas}$ value and the parasitic capacitance causes instability in instrumentation [43]. This technique was applied to a body composition monitoring system used in patients hospitalized in an intensive care unit [41]. The Bode plot without compensation presented errors at high frequencies when the impedance was represented with the Cole–Cole model. The Cole–Cole model considers the electrical representation of biological tissue relating the dielectric function for a liquid or dielectrics with frequency and is commonly used to mathematically represent biological tissue impedance, possessing the CPE element in its mathematical representation as a Cole–Cole impedance function [53]. The Cole impedance function could be obtained from an equivalent two-resistor–one-capacitor circuit by the replacement of the ideal capacitor with a general constant-phase-element (CPE) [54]. In this case, the Cole impedance for the frequency dependence of biological tissue is given by [55]:

(12) $$Z_{Cole}\left(\omega \right)=R_{\infty }+\frac{R-R_{\infty }}{1+{\left(j\omega \tau_{0}\right)}^{1-\alpha }}$$

with $R_{\infty }$ being the resistance of the material at very high frequencies, *j* the imaginary unit and α being a Cole-type distribution of relaxation times, which, for many tissues, is a value in 0.1<α<0.3 [54], and $\tau_{0}$ is the relaxation time [56]. These errors are featured by a sudden increase in the imaginary component. Body impedance presented deviations lower than 2% in comparison with the Cole–Cole model after evaluation of $C_{stray}$, when the correction was added to the measurements [41]. Compensation takes place in cases where $C_{stray}$ values are fixed, just as observed in the previous method. It is essential that one accurately determines the $C_{stray}$ value to avoid the effects of under/over compensation introduced in measurements by Equation (10)’s factor, although it discards the instrumentation’s calibration process for parasitic capacitances.

Among the advantages of this compensation type, one can mention:

- Removal of parasitic capacitance effects on systems where physical removal is not a viable option $C_{stray}$;
- Reduced cost since implementation occurs at the computational level in a processing system;
- Error linked to $C_{stray}$ estimates. When the value is precisely known, then the effect of the parasitic capacitance is fully ruled out, assuming the measurement system is unaffected by the action of $C_{stray}$.

On the other hand, with regard to its disadvantages, one could note that:

- Although it is possible to introduce a parasitic capacitor, based on Equation (11), the dependence on electrode capacitance voltage renders the solution of the system of Equation (11) transcendental, i.e., the system has no analytical solution. Therefore, factor (10) is no longer valid.
- The method critically depends on prior knowledge of $C_{stray}$, since errors associated with its estimate spread errors in $Z_{tissue}$.
- In practical terms, $C_{stray}$ is determined at fixed $f_{meas}$, frequency, which leads to compensation of errors outside the frequency at which $C_{stray}$ is determined. This factor can lead to error propagation in the spectral intervals around $f_{meas}$.

While investigating the effects of electrode pressure on gelatine phantoms in measurements via a 4-point electrode, Dutra [52] noticed the need to elaborate an impedance model to fit the detected peak in impedance modulus at frequencies above 200 kHz. Such an alternative circuit would also enrich the repertoire of parasitic impedance models that would also be useful for creating models and providing compensation to parasitic effects.

### 4.3. Compensation by Negative Impedance Converters

**Figure 5:**
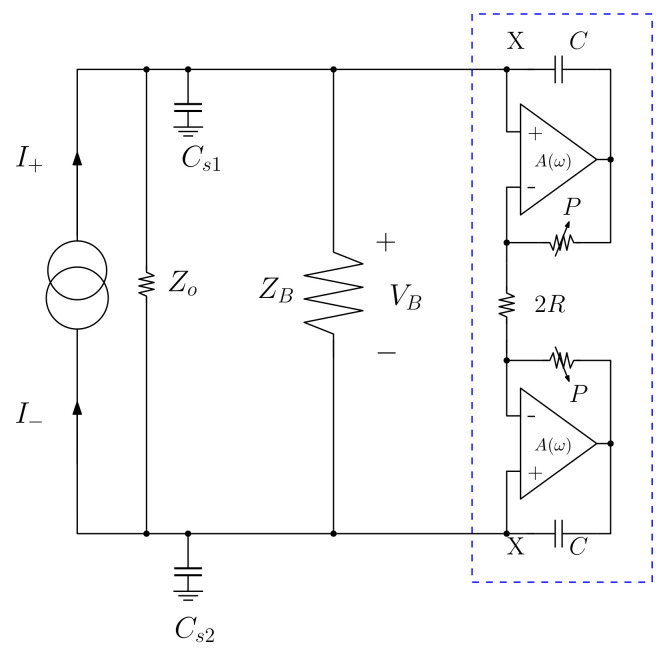
Compensation for NIC use in a tetrapolar EIT. The box highlighted in blue is the negative impedance converter (capacitance). $Z_{B}$, $Z_{o}$, $C_{s1}$ and $C_{s2}$, are the body impedance, output impedance (total) of the current sources $I_{+}$ and $I_{-}$, and the parasitic capacitances of the excitation source, respectively. Source: Adapted from [58].

It is not always possible to gain access to the instrumentation circuits in the measurement systems in EIT. Impedance analyzers are expensive instruments and are often protected by manufacturer warranty or hard internal access. Furthermore, the main contribution from the parasitic capacitance is external to the equipment, since it is observed in cables and connections. Thus, a compensation system that can be remotely added is more suitable. If the parasitic capacitance generated by cables, electrodes, multiplexers, among others, is known, then, an opposite reactance circuit placed in parallel to this capacitance is required. Analog multiplexers in electrical impedance tomography are commonly used. These devices add an additional parasitic loop to the measurement system, which can be approximated by a reactive-capacitive π− filter [57]. If two negative impedance converters (NIC) are placed in parallel to the properly adjusted multiplexer ports, the contributions from these capacitive reactances can be ruled out. The studies presented in [58,59] on EIT are examples that employ such an approach. Figure 5 depicts a diagram of the blocks within an NIC compensation system.

The system in Figure 5 evidences a typical tetrapolar EIT system compensated by NIC. Current sources $I_{+}$ and $I_{-}$ form a symmetric pair that account for exciting the $Z_{B}$ charge. The charge is fluctuating in this configuration in relation to the ground, and allows a configuration that is not dependent on reading and response electrodes [39].

The DC component over the electrodes can distort measurements, lead to electrode polarization and to significant increases in the capacitance seen by the mirrored sources [13]. However, the suppression of $V_{B}$ depends on the sources’ ideal balancing, which is not practical [45]. The error is associated with variations in $V_{B}$, and changes due to frequency, as show in Equation (13). This dependence on frequency poses a barrier for system calibration measurements with fixed values due to an imbalance between sources.

(13) $$V_{B}=\frac{I_{+}-I_{-}}{1/Z_{B}-j\omega C_{s}}$$

The current source I (= $I_{+}$ = $I_{-}$) used in [57], Figure 5, is of the modified Howland type [60]. A coaxial cable transports $I_{+}$ and $I_{-}$ to the $Z_{B}$ charge, such as in a practical situation. When the measurement system is placed close to the tissue, within a situation known as active electrode, then, the parasitic capacitance is the one that forms the instrumentation coat and the electrodes’ construction [13]. In any case, $C_{s1}$ and $C_{s2}$ are observed in the system. The circuit in the highlighted box, which comprises an operational amplifier, R and P resistors, and a capacitor C, forms the NIC. The properly adjusted NIC is capable of nulling the $C_{stray}$ reactance parallel to the transmission line of the $I_{+}$ and $I_{-}$ current, under specific conditions. It is necessary to observe the NIC operation, based on a mesh analysis, to better understand how this effect takes place. The dependence of the operational amplifier of the open loop’s finite gain in frequency can be taken into consideration in order to verify the stability of this methodology.

A routine loop analysis shows that the impedance in the ground’s *X* point, due to NIC, is:

(14) $$Z_{X}=\frac{1}{j\omega C}\left(\frac{1+BA}{1+BA-A}\right)$$

where ω,j,A are the signal’s angular frequency at point *X*, the imaginary unity, and the operational amplifier’s open loop gain, respectively. Term B=R/(R+P) represents the combination of NIC resistors, and this factor’s mitigation in Equation (14) can be experimentally adjusted through *P*. It is worth noting that |B|<1 at all frequencies. If one considers BA≫1 and A≫1 in Equation (14), one finds:

(15) $$Z_{X}=-\frac{1}{j\omega C}\left(\frac{B}{1-B}\right)$$

In other words, NIC behaves as a capacitor whose negative reactance is adjustable by attenuation term *B*. However, *A* is a decreasing function of frequency in the real operational amplifiers. By approximating this dependence through a simple pole located in $\omega_{A}$, the impedance $Z_{X}$ for frequencies higher than the $\omega \gg \omega_{A}$ pole is:

(16) $$\lim_{\omega \to \infty }Z_{X}=\frac{Bj}{\omega C(1-B)}$$

In other words, at high frequencies (understood as higher than the $\omega_{A}$ pole), $Z_{X}$ behaves as an inductor that is dependent on the frequency whose inductance is:

(17) $$L_{x}\left(\omega \right)=\frac{B}{C\omega^{2}\left(1-B\right)}$$

Equation (17) represents an inductor that has a value inversely proportional to the square of frequency, but with a positive value. Thus, there is a risk of having the $L_{X}$ inductance form a resonant circuit with $C_{stray}$, a fact that implies instability. The pole of the current source Io must be dominant over the NIC pole in order to avoid this scenario, given the conditions from which $L_{X}$ emerges. Furthermore, using a Zobel type loop [43] located in $\omega_{A}$ ensures damping of the resonant circuit formed by $C_{stray}$ and $L_{X}$, if it is not enough to ensure such fall. Accordingly, using the Zobel loop allows one to design the sources able to operate at higher resistive charges without losing stability.

Among the advantages of this compensation technique, one finds:

- The parasitic capacitance of cables and multiplexers can be compensated, even when there is no access to the measurement system’s instrumentation;
- Low cost and easy implementation;
- It can be used in any electrode type, be it of excitation or reading.

However, with regard to the disadvantages, it is possible to state that:

- The NIC circuit is only capable of compensating the fixed capacitances. In cases where the parasitic capacitance changes due to frequency and/or voltage, there is a risk of introducing systematic errors to measurements due to over/under compensation;
- Using NIC can trigger instability at high frequencies.

## 5. Discussion and Conclusions

The effects of parasitic capacitance are critical in measurement systems for both impedance and bioimpedance analysis, especially in tomography (EIT) and mainly at high frequencies. Nevertheless, the dynamic nature of electrode capacitance transforms the fixed reactance model into an approximation. Techniques centered on EIT and found in mainstream journals were categorized into three groups, based on the adopted methodology. Overall, these techniques were proposed to compensate for the fixed capacitances, rendering the previous studies essential for the implementation of measurement systems. The chosen compensation methods during measurement processing can emerge as adapted to situations mainly where the parasitic capacitance is somehow dependent on frequency, although the electrode capacitance depends on voltage.

## Data Availability

This study did not report any data.
